# Therapeutic Effects of Coumarins with Different Substitution Patterns

**DOI:** 10.3390/molecules28052413

**Published:** 2023-03-06

**Authors:** Virginia Flores-Morales, Ana P. Villasana-Ruíz, Idalia Garza-Veloz, Samantha González-Delgado, Margarita L. Martinez-Fierro

**Affiliations:** 1 Asymmetric Synthesis and Bio-chemoinformatics Laboratory (LSAyB), Ingeniería Química (UACQ), Universidad Autónoma de Zacatecas, Carretera Zacatecas-Guadalajara Km.6. Ejido la Escondida, Zacatecas 98160, Mexico; 2 Molecular Medicine Laboratory, Academic Unit of Human Medicine and Health Sciences, Universidad Autónoma de Zacatecas, Carretera Zacatecas-Guadalajara Km.6. Ejido La Escondida, Zacatecas 98160, Mexico

**Keywords:** coumarin, antineoplastic, cancer therapy, docking

## Abstract

The use of derivatives of natural and synthetic origin has gained attention because of their therapeutic effects against human diseases. Coumarins are one of the most common organic molecules and are used in medicine for their pharmacological and biological effects, such as anti-inflammatory, anticoagulant, antihypertensive, anticonvulsant, antioxidant, antimicrobial, and neuroprotective, among others. In addition, coumarin derivates can modulate signaling pathways that impact several cell processes. The objective of this review is to provide a narrative overview of the use of coumarin-derived compounds as potential therapeutic agents, as it has been shown that substituents on the basic core of coumarin have therapeutic effects against several human diseases and types of cancer, including breast, lung, colorectal, liver, and kidney cancer. In published studies, molecular docking has represented a powerful tool to evaluate and explain how these compounds selectively bind to proteins involved in various cellular processes, leading to specific interactions with a beneficial impact on human health. We also included studies that evaluated molecular interactions to identify potential biological targets with beneficial effects against human diseases.

## 1. Introduction

The medicinal use of compounds that are derived from natural sources such as fungi, plants, and animals precede documented human history as an ancient tradition by perhaps thousands of years [1]. These compounds have often been used to treat diseases and injuries with an important role in drug discovery and drug development thanks to the modern tools of molecular biology, biochemistry, and chemistry, which allow us to dissect biological effects on the human body, as well as possible interactions [2]. These advances put natural compounds in the spotlight as potential new therapies against many complex diseases, including inflammatory, vascular, hypertensive, infectious, and neurodegenerative diseases, as well as cancer [3].

Among the most studied natural compounds in human health are coumarins, which are molecular compounds of natural and synthetic origin. They constitute a family of heterocyclic compounds and have been extensively studied in the biochemical and pharmaceutical fields [4]. In the following paragraphs, we provide a narrative revision of the origin of the coumarins, their chemical classification, and their pharmacological activities, which position them as potential therapies for diseases such as cancer, diabetes mellitus, cardiovascular, neurodegenerative, infectious, and inflammatory diseases, among others. We include an emphasis on cancer, as conventional therapies induce side effects in patients, impacting their quality of life. The use of synthetic or naturally occurring compounds has opened new ways to targeted and personalized therapies by affecting signaling pathways in which therapeutic targets are the main objective.

## 2. Coumarins 

**Figure 1:**
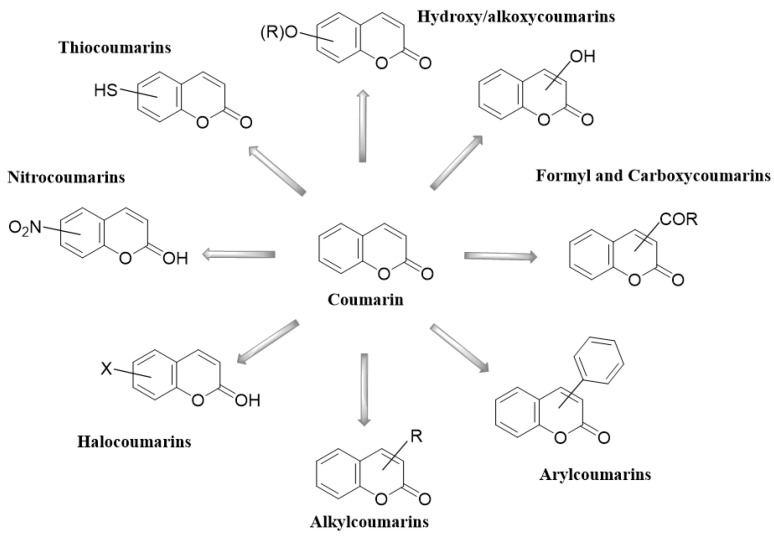
Coumarin as a basic nucleus for obtaining simple coumarins. Taken from Borges, F. et al. (2005) [6]. Accessed date 28 November 2022.

Coumarins have been studied for more than 200 years. Their name derives from the species *Coumarouna odorata* Aube (*Dipteryx odorata*), from which they were isolated for the first time [5]. Coumarin is a secondary metabolite found naturally in various plant families and in essential oils. The basic nucleus of coumarin (Figure 1) corresponds to the compound benzo-a-pyrone (2*H*-1-benzopyran-2-one), whose nomenclature was established by the International Union of Pure and Applied Chemistry (IUPAC). In addition to the isolated coumarins of natural origin, the synthetic derivatives due to the substituents at several positions in the chemical structures have increased the number of coumarins currently known, with interesting biological effects [1,5].

The coumarin 2*H*-chromen-2-one, 1,2-benzopyrone, being a semi-volatile lactone of low molecular weight, has a sweet odor and is present in more than 60 plants [7]. It was also identified that coumarins are present in tobacco, but in relatively low amounts, something that explains the cytotoxic of tobacco toxicants [8]. 

Coumarins and their metabolites have been useful in the pharmaceutical industry; they are part of the basic nucleus of many compounds with therapeutic purposes due to the great potential they have. Coumarins are present in various types of plants, among which we can mention *Rutaceae*, *Apiaceae*, *Asteraceae*, *Leguminoceae,* and *Thymelaeaceae*. Among the various therapeutic effects that have been identified, it was found that they present antimicrobial, antiarrhythmic, anti-HIV, and antineoplastic activity. Recent research has described the structure and biological function of analogues derived from coumarins synthesized from plants. Coumarins are classified into six main groups: simple coumarins [9], furanocoumarins [10], dihydrofuranocoumarins [11], pyranocoumarins [12], phenylcoumarins [13], and biscoumarins [14]. The six types of coumarins are shown in Table 1, showing some characteristic examples.

The different derivatives of coumarins have characteristic properties that allow them to possess diverse therapeutic effects and therefore multiple applications in several areas of medicine showing to be useful as anticancer, carbonic anhydrase inhibitors, anti-HIV, anti-inflammatory, and anticoagulant agents, as biomarkers, and in green chemistry [43,44]. 

One of the main factors to which the biosynthesis of coumarin derivatives has been resorted is the resistance generated by the administration of some drugs, which has become a great inconvenience for the development and treatment of different types of cancer. Derived from this, compounds of natural origin and certain synthetic molecules to assess the possible resistance generated at the time of administration, have demonstrated significant potency in the inhibition of multidrug resistance (MDR) in patients with cancer [45]. 

## 3. Coumarins and Their Pharmacological Activity

Coumarins present diverse biological activities derived from their substitution pattern, which has an impact on their physicochemical properties and pharmacological application [46]. Crum-Brown and Frasser, through their research, proposed a theory by which they demonstrated that the mechanism of action and the pharmacological effect of a substance depends on the chemical composition [47]. 

**Figure 2:**
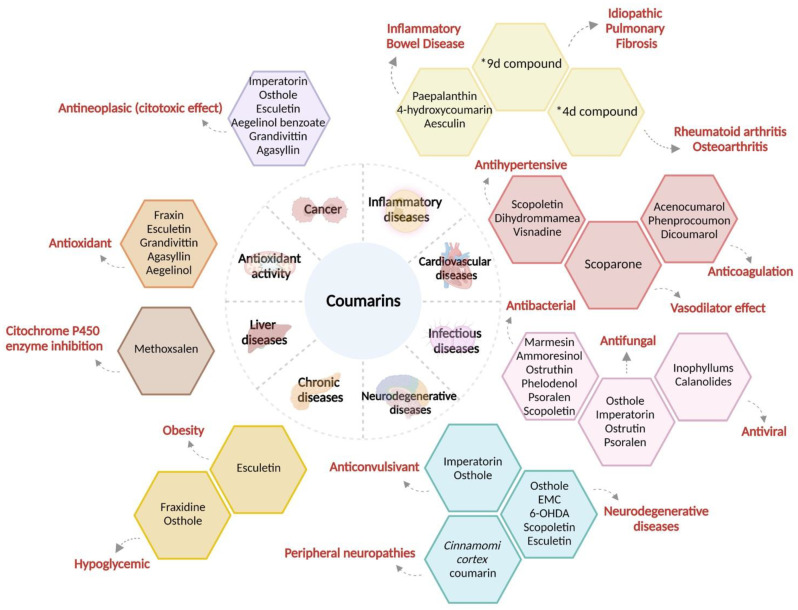
Properties and pharmacological effects of coumarins and coumarin derivatives against human diseases. A wide range of effects and pharmacological properties of cumarins have been reported; as we can see, scopoletin, esculetin, and osthole have been the most described coumarins to be involved in the modulation of different systems such as cardiovascular and neurological system. A great knowledge of coumarins is their variety number of antineoplastic properties, acting as cytotoxic compounds in different kinds of cancer. *9d compound: (*N*-(4-((2-((2-oxo-2*H*-chromen-4-yl)oxy)phenyl)-2-(piperidin-1-yl)acetamide); *4d compound: 3-(1*H*-benzo[*d*]imidazol-2-yl)-6-chloro-2*H*-chromen-2-one.

Coumarins have shown hepatic metabolism where cytochrome P450 acts as an inducer of the oxidative processes of coumarins. It has also been demonstrated that the intestinal microbiome plays an important role. Coumarins are rapidly absorbed and distributed throughout the body with high concentrations detected in the liver and kidneys. Significant tissue accumulation of coumarin or its metabolites by the oral route has not been determined. The route of elimination depends on the administration and type of coumarin [48]. The main therapeutic effects of coumarins are described in the following paragraphs (Figure 2).

### 3.1. Therapeutic Use of Coumarins in Inflammatory Diseases

**Figure 3:**
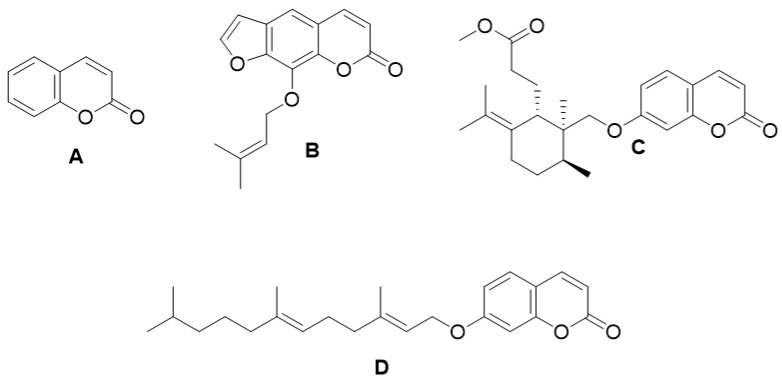
Chemical structures of coumarin (**A**), imperatorin (**B**), methyl galbanate (**C**), and umbelliprenin (**D**).

Coumarin (Figure 3A) has shown a therapeutic effect against edema, eliminating proteins and fluid from injured tissue by activating mechanisms such as phagocytosis, enzyme release and proteolysis [49]. Research in the field of pharmacology has indicated that imperatorin (Figure 3B) has an anti-inflammatory effect in lipopolysaccharide-stimulated mouse macrophages (RAW264.7) in an in vitro model of edema, as it inhibits the protein expression of nitric oxide synthase (NOS) and cyclooxygenase-2 (COX-2) [50]. Rabe et al. identified the anti-inflammatory and immunomodulatory effect of terpenoid coumarins (umbelliprenin and methyl galbanate) (Figure 2C,D) in vivo and in vitro demonstrating proliferative effects and the release of interleukin 4 (IL-4) and interferon (INF)-γ upon COX-2 inhibition [51,52].

#### 3.1.1. Coumarins in Inflammatory Bowel Disease

Inflammatory bowel disease (IBD) is considered a non-contagious pathology presenting chronic inflammation of the intestine, classified as Crohn’s disease or ulcerative colitis, which still has no definitive treatments or cure. The etiology of this disease is still not entirely clear, which is a limiting factor for the development and synthesis of new drugs; therefore, treatments are scarce. Some new pharmacological approaches have been directed towards the use of coumarins and their derivatives because of their antioxidant and anti-inflammatory effects. These compounds are useful as complementary therapy in IBD, as they modulate signaling pathways that generate protective effects against intestinal inflammation and oxidative stress (OS), characteristic aspects of the disease [53]. 

**Figure 4:**
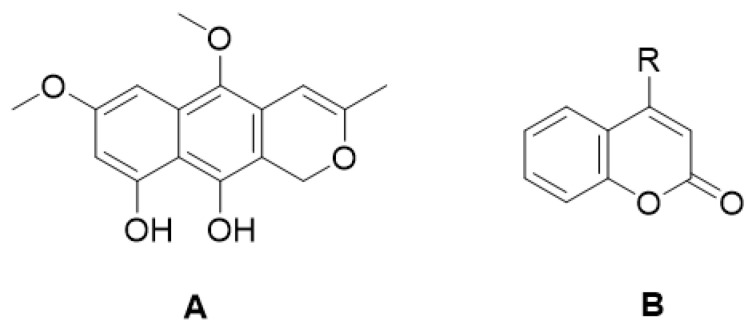
Chemical structure of paepalanthin (**A**) (9,10-dihydroxy-5,7-dimethoxy-1*H*-naptho(2,3c)pyran-1-one) and coumarin (R=H) and 4-hydroxycoumarin, R=OH (**B**).

Paepalanthin (9,10-dihydroxy-5,7-dimethoxy-1*H*-naptho(2,3c)pyran-1-one) (Figure 4A) is a coumarinic derivative synthesized from the Brazilian endemic plant *Paepalanthus bromelioides* of the botanical family *Eriocalulaceae*. It was the first isocoumarin tested in an experimental murine model of intestinal inflammation, and it demonstrated protective effects in the acute and relapse phases of 2,4,6-trinitrobenzene sulfonic acid (TNBS)-induced intestinal inflammation in rats [54]. Another study evaluated the activity of 4-hydroxycoumarin (Figure 4B) in the acute and subchronic phases of the TNBS-induced intestinal inflammation in rats, with significant inhibition of tissue damage and injury observed [55].

**Figure 5:**
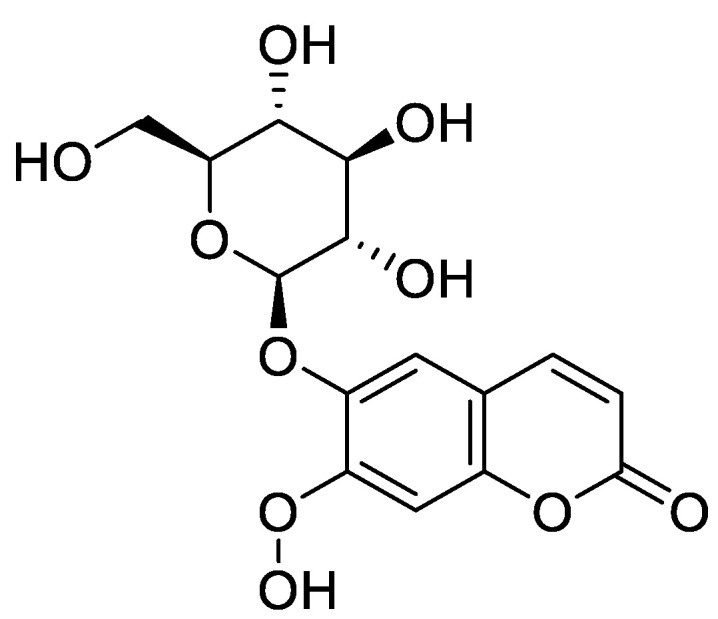
Chemical structure of aesculin (7-hydroxy-6-*O*-glucosylcoumarin).

Aesculin (7-hydroxy-6-*O*-glucosylcoumarin) (Figure 5), another coumarin derivative, demonstrated protective effects on dextran sulfate sodium (DSS)- and TNBS-induced intestinal inflammation by counteracting glutathione (GSH) depletion and inhibiting myeloperoxidase (MPO) activity, thereby suppressing clinical indicators of intestinal inflammation and histopathological damage promoted by DSS. The effects were accompanied by the downregulation of inducible NOS (iNOS) expression. In vitro studies performed with LPS-stimulated RAW264.7 cells showed that esculin reduced NO generation as well as iNOS gene expression and protein levels [56].

#### 3.1.2. Coumarins in Idiopathic Pulmonary Fibrosis 

**Figure 6:**
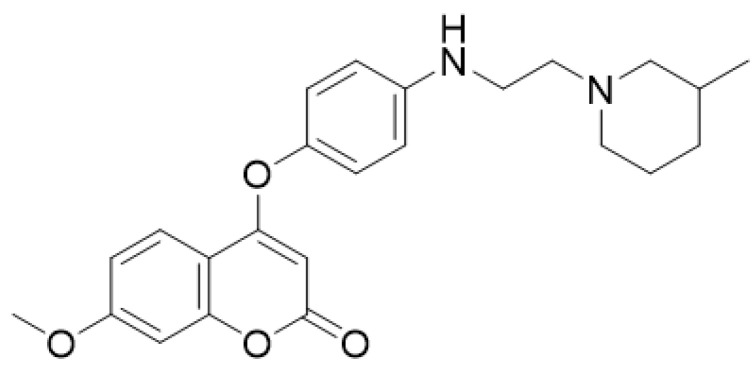
Chemical structure of *N*-(4-((2-((2-oxo-2*H*-chromen-4-yl)oxy)phenyl)-2-(piperidin-1-yl)acetamide.

Idiopathic pulmonary fibrosis, characterized by excess accumulation of extracellular matrix, is involved in many chronic diseases or injuries and threatens human health greatly. Inflammation, due to systemic and local responses of living tissues to injury, also plays an important role in the development of fibrosis. Currently, there are very few small molecule compounds approved for the treatment of fibrotic diseases. So far, only pirfenidone and nintedanib have been approved by the United States Food and Drug Administration (FDA) for the treatment of IPF [57,58]. The design of a new series of compounds with antifibrotic effects by hybridization of coumarin and the hydrophobic group of nintedanib, demonstrated that compound (*N*-(4-((2-((2-oxo-2*H*-chromen-4-yl)oxy)phenyl)-2-(piperidin-1-yl)acetamide) (Figure 6) of the 30 compounds synthetized showed potent antifibrotic abilities in inhibiting TGF-β-induced total collagen accumulation in NRK-49F cells with low toxicity and macrophage migration. Authors also observed that the coumarin suppresses TGF-β1-induced protein expression of COL1A1, α-SMA, and p-Smad3. Based on the demonstrated effects and low toxicity, it has been considered a promising and potentially orally active candidate for the treatment of fibrotic disease [59].

#### 3.1.3. Coumarins in Rheumatoid Arthritis and Osteoarthritis

**Figure 7:**
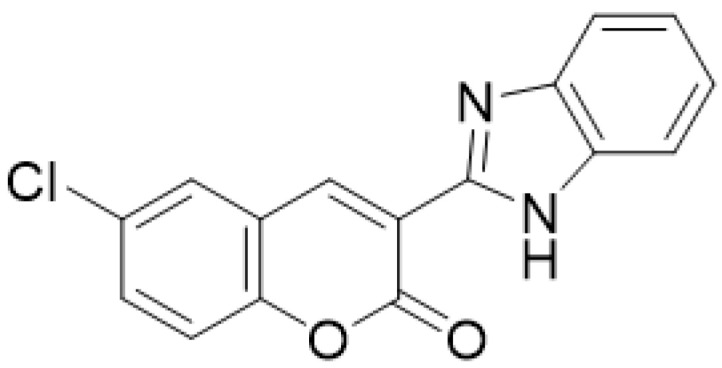
Chemical structure of 3-(1*H*-benzo[*d*]imidazol-2-yl)-6-chloro-2*H*-chromen-2-one.

There are a number of diseases in which the main pathological condition is an inflammatory process, among which are rheumatoid arthritis, osteoarthritis, and some others, such as gout and, even, obesity. Therefore, inhibition of the inflammatory process is of vital importance in treatment. First-line non-steroidal anti-inflammatory drugs (NSAIDs) such as indomethacin, ibuprofen and naproxen are useful for chronic inflammatory diseases. However, these treatments have been shown to generate gastrointestinal complications despite a good anti-inflammatory effect [60]. Derivatives of natural origin, such as coumarins, have been shown to have anti-inflammatory effects, with structure-activity relationship (SAR) results showing that if an aromatic group binds directly in position 3 of the basic nucleus of coumarin, the molecule shows anti-inflammatory activity [61]. An example is provided in a study carried out in 2014, in which two series of coumarin-benzimidazole derivatives were synthesized and evaluated for their anti-inflammatory activity. The results showed that compound 3-(1*H*-benzo[*d*]imidazol-2-yl)-6-chloro-2*H*-chromen-2-one (Figure 7) was the ideal candidate for the development of new gastric mucosal protective anti-inflammatory agents, useful for the treatment of rheumatoid arthritis and osteoarthritis [62].

### 3.2. Coumarins in Cardiovascular Disease

Coumarin derivatives have demonstrated pharmacological effects based on the substituents, e.g., coumarins with different heterocycles based on the cyclization of 2-ethoxy-3-phenylpropanoic acid and 2-benzylmalonic acid have been investigated as lipid-lowering agents to inhibit the formation of atheroma caused by the accumulation of triglycerides and cholesterol in the walls of arteries or blood vessels, thereby decreasing the incidence of cardiovascular disease [63]. Coumarin 7,8-dihydroxy-3-(4-methylphenyl)coumarin was assessed for its lipid-lowering effects in a murine model of hypercholesterolemia, and led to a significant reduction in serum cholesterol levels [64]. 

#### 3.2.1. Coumarins with Antihypertensive Activity 

**Figure 8:**
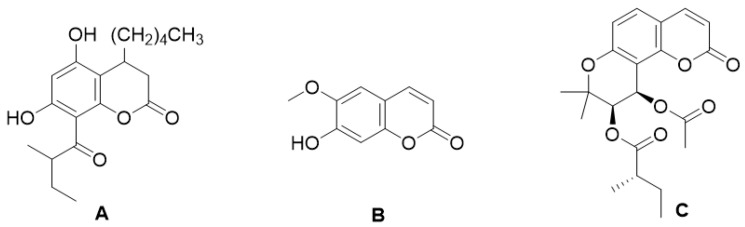
Chemical structures of dihydrommamea (**A**), scopoletin (**B**), visnadine (**C**).

Certain coumarin derivatives such as dihydrommamea (Figure 8A), a new coumarin synthesized from the seeds of the West African tree *Mammea africana* (Sabine), have demonstrated antihypertensive effects through a series of experiments using methanol and dichloromethanol extracts of the stem bark tested in a murine hypertension model in Wistar rats, in which a therapeutic effect was demonstrated [65]. Another coumarin, scopoletin (Figure 8B), isolated from the fruits of *Tetrapleura tetraptera* (Mimosaceae), showed a hypotensive effect in murine in vivo and in vitro models due to its smooth muscle relaxant activity [66]. Visnadine (Figure 8C), a coumarin isolated from the fruit of *Ammi visnage*, has vasodilator and coronary therapeutic effects and has been very useful in treating angina pectoris [67].

#### 3.2.2. Coumarins with Vasodilator Effect

**Figure 9:**
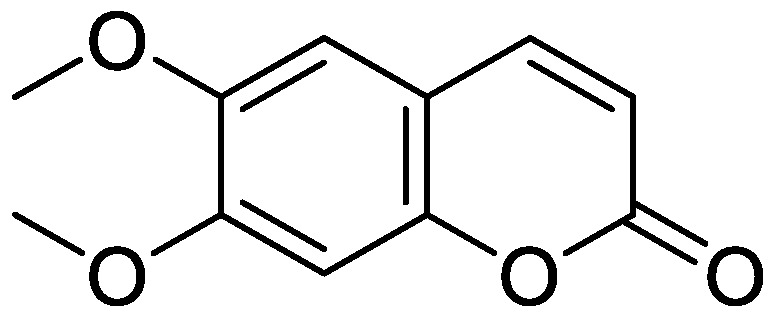
Chemical structures of scoparone (6,7-dimethoxy coumarin).

Vasoconstriction is a condition in which the blood vessels are narrowed due to the musculature of the vascular walls. When blood vessels constrict, the blood circulation becomes sluggish or blocked. Huang et al. investigated the vasodilator mechanisms of scoparone (6,7-dimethoxy coumarin) (Figure 9), a coumarin derivative, which was shown to dilate rat aortic rings precontracted with phenylephrine in a dose-dependent manner; this compound also led to a significant reduction in plasma lipoproteins in hyperlipidemic rabbits. It was concluded that scoparone inhibits the mechanism of action of agonists by acting on calcium-dependent channels and depressing the contractile response in aortic rings. The results suggested that scoparone may be a promising agent for the development and synthesis of new immunosuppressants with vasodilatory action [68,69].

#### 3.2.3. Coumarins in Heart Failure

**Figure 10:**
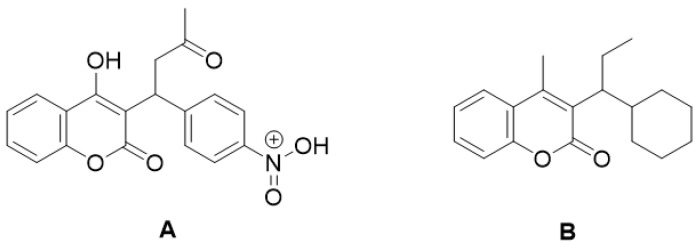
Chemical structures of anticoagulants. acenocoumarol (**A**), phenprocoumon (**B**).

Heart failure is a condition in which the heart can no longer pump oxygen-rich blood to the rest of the body efficiently. This causes symptoms throughout the body. It has been determined that heart failure is a risk factor for the administration of coumarin anticoagulants. Therefore, Visser et al. conducted an investigation aimed at identifying whether patients with heart failure present over-anticoagulation with acenocoumarol and phenprocoumon (Figure 10) in a cohort study. They included 1077 patients taking coumarin anticoagulants. As a result, it was found that heart failure is an independent risk factor for excessive anticoagulation, since 396 of the patients had an international normalized ratio (INR) greater than or equal to 60, so that patients with heart failure should be monitored and their possible reactions to internal bleeding should be assessed [70,71].

#### 3.2.4. Coumarins for the Treatment of Thromboembolic Conditions

**Figure 11:**
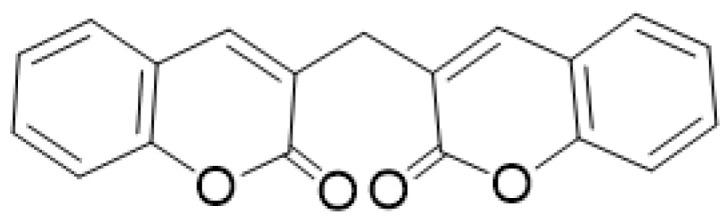
Chemical structure of dicoumarol.

One of the coumarins that has been shown to have anticoagulant activity is dicoumarol, which belongs to the biscoumarin classification (Figure 11). It has been shown to be a selective vitamin K antagonist involved in coagulation processes and hemorrhage formation. Coagulation factors II, VII, IX, and X require a carboxylation process to be activated by the cofactor vitamin K; this is responsible for the therapeutic effect of dicoumarol as an anticoagulant [72].

### 3.3. Coumarins in Infectious Diseases

Coumarins with antimicrobial effects has been evaluated in several reports. They may have anti-bacterial, antifungal, and/or antiviral activities, as described in the following sections.

#### 3.3.1. Antibacterial Activities of Coumarins

**Figure 12:**
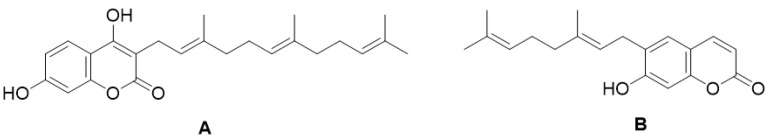
Chemical structures of ammoresinol (**A**) and ostruthin (**B**).

Simple coumarins have shown antibacterial activity derived from hydrocarbon substitutions in the basic coumarin nucleus. Ammoresinol (Figure 12A) and ostruthin (Figure 12B) have shown therapeutic activity against a large number of Gram+ bacteria such as *Bacillus megaterium*, *Micrococcus luteus*, and *Staphylococcus aureus* [73]. Another synthetic coumarin derivative, 2,2-dimethylpyranocoumarin was shown to be antibacterial against a broad spectrum of bacteria, inhibiting their metabolism and their mechanisms of reproduction and protein synthesis [74]. 

A major current problem is the emergence of bacterial resistance, so the synthesis of new classes of antimicrobial agents with a potential to target certain types of microorganisms has become a key objective in pharmacological research. Thus, coumarins have become a promising class of bioactive heterocyclic compounds [75]. 

**Figure 13:**
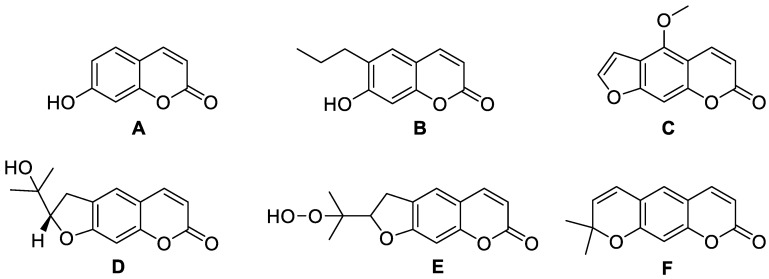
Chemical structures of umbelliferone (**A**), phelodenol (**B**), bergaptene (**C**), marmesin (**D**), rutaretin (**E**), and xanthyletin (**F**).

Umbelliferone and other coumarin derivatives such as phelodenol, psoralen, scopoletin, bergaptene, marmesin rutaretin, and xantholein were isolated from the plant *Fatoua pilosa* and shown to be active against *Mycobacterium tuberculosis*, especially umbelliferone (Figure 13A) and scopoletin (Figure 8B) [76,77].

#### 3.3.2. Coumarins with Antifungal Activities

**Figure 14:**
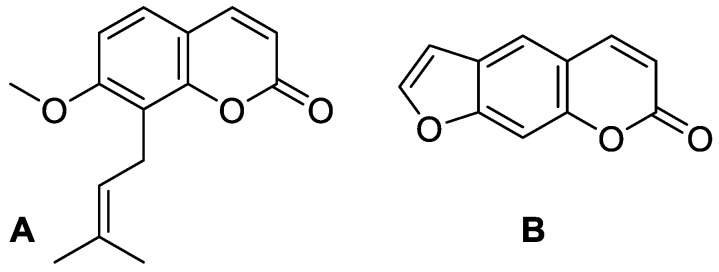
Chemical structured of osthole (**A**) and psoralen (**B**).

One of the coumarin derivatives that has shown an antifungal effect is osthole (Figure 14A), which was extracted from the plant *Angelica pubescens*. This compound was found to have an antifungal effect against several pathogens present in plants, such as *Rhizoctonia solani, Phytophtora capsici, and Botrytis cinera*, inhibiting bacterial growth through their signaling pathways at the metabolic and molecular levels [78]. Tests have been carried out to identify which coumarin derivatives have antifungal effects, imperatorin, ostrutin, and psoralen showing the most promising effects (Figure 14B) [79].

#### 3.3.3. Coumarins with Antiviral Activity 

**Figure 15:**
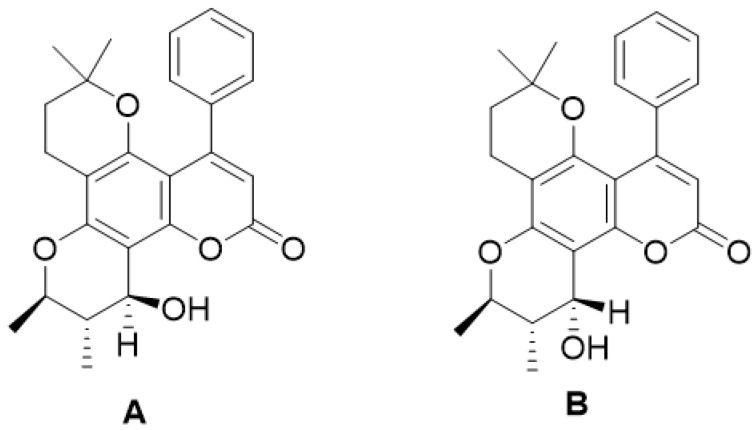
Chemical structured of inophillums B (**A**) and inophillums P (**B**).

Many derivatives of natural origin have shown antiviral activities, including the coumarins, but the antiviral therapeutic effect depends on the substituent. Some of these derivatives have been tested against HIV [80]. In the same way, new coumarin derivatives such as inophyllumsand calanolides isolated from the giant African snail *Achatina fulica* have shown antiviral effect. Inophyllums B and P inhibited reverse transcriptase (RT) in HIV in cell culture, selectively against HIV-1 (Figure 15) [81].

### 3.4. Coumarins in Neurodegenerative Diseases

Neurodegenerative diseases are a heterogeneous group of disorders that are characterized by the progressive degeneration of the structure and function of the central nervous system or peripheral nervous system, and are an important public health problem in older adults. This group of diseases includes Alzheimer’s disease [82], Parkinson’s disease and Huntington’s disease, among others. The greatest known risk factor for many neurodegenerative disorders is age. These figures are likely to rise as the population ages, making neurodegenerative disorders a growing healthcare concern. Coumarins are considered to be promising molecules against several neurodegenerative diseases because they have been shown to ameliorate clinical manifestations and symptoms, as described in the following sections.

#### 3.4.1. Coumarins with Neuroprotective Activity 

Research in the area of pharmacology has shown that esculetin has neuroprotective effects as well as anti-inflammatory activity in ischemic lesions and cerebral perfusion, demonstrated in a murine model of cerebral artery occlusion by administering 20 mg/mL esculetin intracerebroventricularly 30 min before ischemia occurred [83]. 

#### 3.4.2. Epilepsy

One of the coumarins shown to have anticonvulsant activity for the treatment of epileptic seizures is imperatorin (Figure 3B), which was tested in a mouse model with median effective dose (ED_50_) values of 167 and 290 mg/kg [84,85]. Another coumarin derivative that also exhibits anticonvulsant effects is osthol (Figure 14B), showing ED_50_ values ranging from 253 to 639 mg/kg where the neurotoxic effects were more noticeable [86]. 

#### 3.4.3. Multiple Sclerosis 

Multiple sclerosis (MS) is the most common demyelinating disease, in which the insulating covers of nerve cells in the brain and spinal cord are damaged. This damage disrupts the ability to transmit signals in the nervous system, resulting in a range of signs and symptoms, including physical, mental, and sometimes psychiatric problems.

Osthole (Figure 14B), a coumarin derivative, has been found to be effective against MS. The therapeutic effects of osthole on demyelination in the central nervous system were tested in a murine model of autoimmune encephalomyelitis and MS. The results showed that osthole retarded the disease process when the therapy was initiated in the subclinical period, suggesting that osthole might be a new pharmacological approach to treating MS [87,88].

#### 3.4.4. Parkinson’s Disease

**Figure 16:**
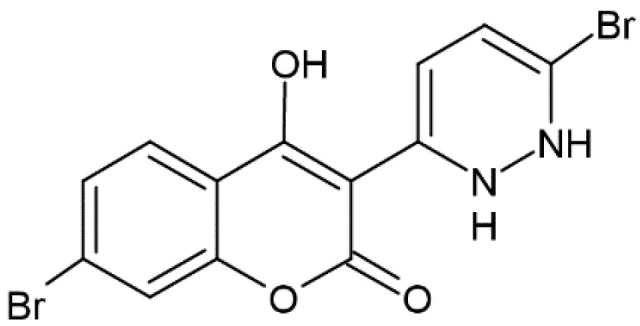
Chemical structure of 7-bromo-3-(6-bromopyridazine-3yl) coumarin.

Parkinson’s disease (PD) is a disorder of the nervous system. It results from damage to the nerve cells that produce dopamine, a chemical that is vital for the smooth control of muscles and movement. Research was carried out in which Enriquez and collaborators tested coumarin derivatives for PD. Among them, the 3-pyridazinylcoumarin scaffold was reported as the base nucleotide for reversible and selective inhibition against MAO isoforms. The objective of the research was focused on the hybrid synthesis of coumarin-pyridazine compounds which were tested on MAO-A and MAO-B isoforms directly affecting the MAO-B isoform. The compound 7-bromo-3-(6-bromopyridazine-3yl)coumarin (Figure 16) had the highest effectiveness with an IC_50_ of 60 nM and no cytotoxic effects at the neuronal level. This compound was tested in an in vivo mouse model of reserpine-induced Parkinsonian disease and supported by molecular docking analysis, showing that it is a promising antiparkinsonian candidate [88]. 

**Figure 17:**
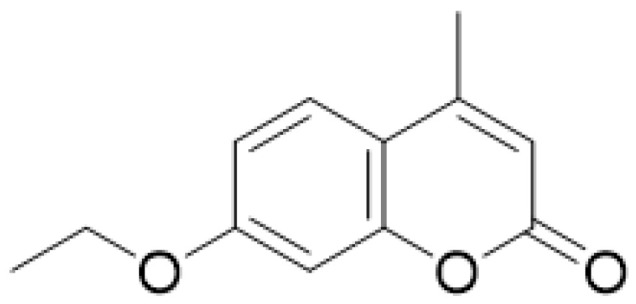
Chemical structure of 7-ethoxy-4-methylcoumarin (EMC).

Another study conducted by Li et al. investigated the possible effect of the compound 7-ethoxy-4-methylcoumarin (EMC) (Figure 17) on PD through a murine mouse model induced by 1-methyl-4-phenyl-1,2,3,6-tetrahydropyridine (MPTP) and in the nematode *C. elegans* exposed to 6-hydroxydopamine (6-OHDA). The study showed that EMC had an agonist effect against the dopamine D2 receptor (DRD2), significantly improving motor areas and the clinical manifestations of PD. Thus, these findings suggest that EMC may be beneficial for PD patients [89].

#### 3.4.5. Amyotrophic Lateral Sclerosis

Amyotrophic lateral sclerosis (ALS) is a disease known as Charcot or Lou Gehrig’s disease considered a progressive neurodegenerative disorder affecting motor areas. This disease is classified depending on the clinical manifestation as: familial (fALS) with 10% recurrence, inherited autosomal dominant, autosomal recessive, X-linked, and sporadic (sALS) with 90% recurrence without a genetic load. Both have similar clinical manifestations among which we can mention muscle weakness, spasms, and cramps; in a more advanced stage, dyspnea, dysphagia, paralysis, and even, death are manifested in a period of 3 to 5 years of onset. Its incidence is associated with adults between 60 and 65 years of age [90]. 

To evaluate the potential effect of coumarins against neurodegenerative diseases, Morgan and collaborators using in vivo models evaluated scopoletin (Figure 8B) synthesized from *Canarium patentinervium*. Authors demonstrating an antioxidant, anti-inflammatory effect and presenting a moderate anticholinesterase activity, becoming a promising scaffold for the synthesis and development of targeted drugs against neurodegenerative diseases such as ALS [91].

**Figure 18:**
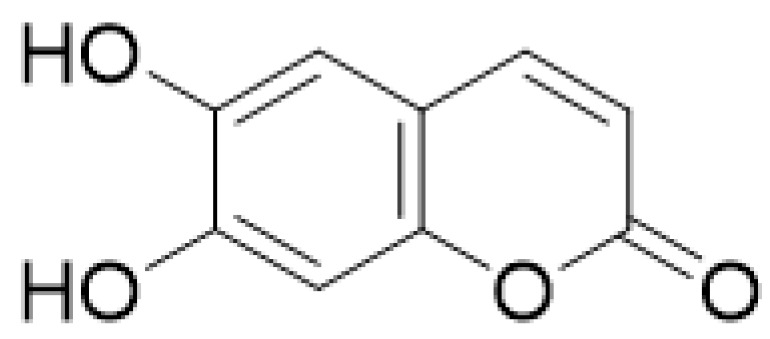
Chemical structure of esculetin.

Siân C. Barber et al. tested the effect of esculetin (Figure 18), a coumarin derivative administered in a murine model in which OS was induced in rat NSC34 motor neurons, showing that it inhibits the activation of the NF-κB signaling pathway and 5-LOX involved in neurodegenerative diseases, being a promising molecule [92].

#### 3.4.6. Alzheimer’s Disease

Alzheimer’s disease (AD) is a neurodegenerative disease in which the main clinical manifestation is dementia, characterized by a progressive, multifactorial, and fatal nature. AD is considered a major public health problem worldwide and is the fourth leading cause of death after cardiovascular disease and cancer. The main cause of AD is conditioned by a deficiency in brain levels of the neurotransmitter acetylcholine (ACh), which is hydrolyzed by acetylcholinesterase (AChE) [93]. 

**Figure 19:**
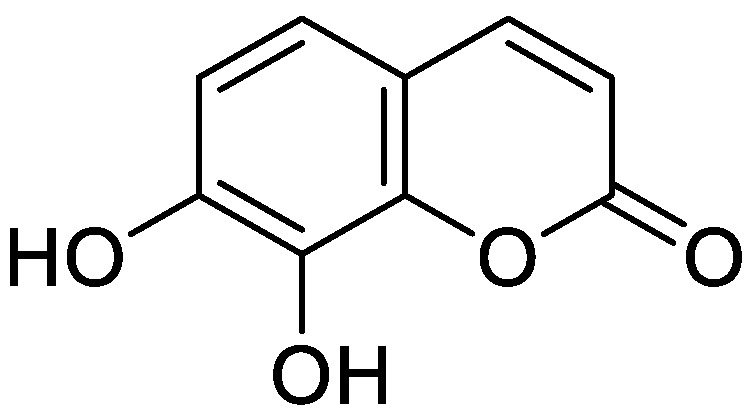
Chemical structure of daphnetin (7,8-dihydroxycoumarin).

A study conducted by Ali et al. carried out a structure-activity analysis to identify the possible anti-Alzheimer’s disease (anti-AD) activity of coumarin derivatives isolated from *Angelica decursiva* and *Artemisia capillaris* compared to the reference coumarin daphnetin (Figure 19). The study demonstrated the inhibitory effect of umbelliferone 6-carboxylic acid, esculetin and daphnetin exhibited potent inhibitory activity against acetylcholinesterase (AChE), butyrylcholinesterase (BChE), and amyloid β-site 1 protein (BACE1). On the same way, molecular docking showed that umbelliferone 6-carboxylic acid (Figure 13A) esculetin (Figure 18) and daphnetin, different residues of BACE1 interacted with hydroxyl and carboxylic groups, and the binding energies of umbelliferone 6-carboxylic acid, esculetin and daphnetin were negative. Based on structure–activity relationships of these coumarins, authors speculated that the presence of a free hydroxyl group (catechol) at the C-6, 7, and 8 positions plays a predominant role in AChE and BChE inhibition, while the presence of a carboxyl and catechol group plays a crucial role in BACE1 inhibition. According to the above, authors suggested umbelliferone 6-carboxylic acid, esculetin and daphnetin have anti-AD effects by inhibiting AChE, BChE, and BACE1, which might be useful against AD [94].

#### 3.4.7. Huntington’s Disease

Huntington’s disease (HD) is characterized as a hereditary neurodegenerative disorder that causes the wasting of nerve cells caused by an abnormal expansion of the CAG trinucleotide repeat in exon 1 of the huntingtin gene (HTT), which leads to mutation of the HTT protein through various signaling mechanisms, resulting in neuronal death. The clinical manifestations of this disease are motor dysfunction, cognitive deficits, compromised daily living capacity and brain neurodegeneration [95].

The therapeutic effect against HD of esculetin (Figure 18), a coumarin derivative with possible neuroprotective effects, was tested by Pruccoli et al. In their study, the authors demonstrated that, in a PC12-inducible *Drosophila melanogaster* HD transgenic model expressing fragments of the mutated HTT protein (mHTT) esculetin decreased OS and mitochondrial deterioration generated by mHTT and inhibited cell death [96].

#### 3.4.8. Peripheral Neuropathies

Peripheral neuropathy refers to the many conditions that involve damage to the peripheral nerves causing weakness, numbness, and pain, usually in the hands and feet, but also impacting the function of the digestive system, kidneys, and circulation. Glial cells have been found to play an important role in neuropathic pain, whereby these cells release various nociceptive mediators, including proinflammatory cytokines such as interleukin 1-beta (IL-1β) and tumor necrosis factor (TNF). 

In an investigation conducted by Kim et al., the effect of coumarin present in the aqueous extract from *Cinnamomi cortex* was tested to determine whether it attenuated cold allodynia induced by oxaliplatin, a chemotherapeutic drug that induces acute peripheral neuropathy characterized by cold allodynia after administration. The therapeutic effect of coumarin was tested in a murine rat model in which oxaliplatin (6 mg/kg, i.p.) was administered in a single dose and induced significant signs of cold allodynia. Treatment significantly inhibited the activation of astrocytes and microglia, as well as the expression levels of IL-1β and TNF in the spinal cord, demonstrating that coumarin has a potent anti-allodynic effect [97]. 

### 3.5. Coumarins in Chronic Degenerative Diseases

A degenerative disease is a disease in that in which the function or structure of affected tissues or organs worsens over time. The pharmacological properties of coumarins have also been tested with the aim of lessening the signs and symptoms of degenerative diseases, including the complications of obesity [98], metabolic syndrome [99,100], and type 2 diabetes mellitus (T2DM) [101,102] among others, as described in the following sections.

#### 3.5.1. Obesity

The coumarin derivative esculetin was shown to have a therapeutic anti-adipogenic effect tested on the preadipocyte cell line 3T3-L1 in an in vitro assay, showing significant inhibitory activity against adipocyte differentiation by analyzing fat accumulation [98].

#### 3.5.2. Metabolic Syndrome

Metabolic syndrome is characterized by the presence of a series of physiological, biochemical, metabolic, and even, clinical abnormalities, and is closely linked to overweight, obesity and lack of physical activity. It is the main risk for the development of cardiovascular disease and T2DM [99].

T2DM is one of the most important diseases in developed nations. This metabolic disorder is characterized by a high level of blood glucose and usually causes serious damage to the eyes, kidneys, and cardiovascular system if not immediately treated. Several factors are reportedly involved in the appearance of high glucose levels. They include insulin resistance, pancreatic cell dysfunction, and excess hepatic glucose production [100].

#### 3.5.3. Coumarins with Antihyperglycemic Activity in Diabetes Mellitus

**Figure 20:**
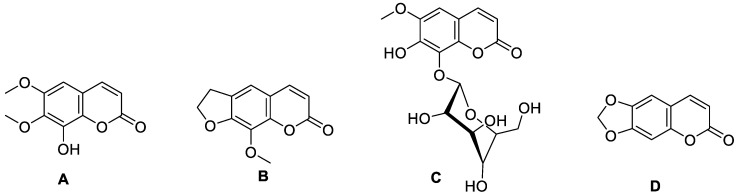
Chemical structures of fraxidine (**A**), methoxsalen (8-methoxypsoralen) (**B**), fraxin (**C**) and, ayapin (6,7-methylenedioxycoumarin) (**D**).

The coumarin derivative, fraxidine (Figure 20A), synthesized from the plant *Teramnus labialis* (Fabaceae), showed an antihyperglycemic effect in a C57BL/Ks-db/db murine model of T2DM [101].

A study was conducted in which Liang et al. tested the coumarin derivative osthole (7-methoxy-8[3-methylpent 2-enyl]coumarin) (Figure 14B) synthesized from *Cnidium monnieri* (L.) Cusson and *Angelica pubescens* to investigate the hypoglycemic effect in a murine model of diabetic db/db mice and in vitro assays. It was demonstrated that osthole generated direct inhibition towards peroxisome proliferator-activated receptors (PPAR), which are steroid transcription factors that affect the expression of multiple target genes involved in glucose metabolism and fatty acid storage, suggesting that this compound is effective for the treatment of hyperlipidemia, hyperglycemia and insulin resistance. Obese diabetic db/db mice were treated with osthole by different routes of administration, and osthole was found to significantly reduce blood glucose levels. Therefore, the in vitro and murine model results have shown that osthole could be a dual PPAR inhibitor, significantly alleviating hyperglycemia, making osthole a promising new drug for the treatment of diabetes mellitus [102].

### 3.6. Coumarins in Liver Diseases

A coumarin derivative that showed a microsomal inhibitory effect against the cytochrome P450 enzyme was methoxsalen (8-methoxypsoralen) (Figure 20B) synthesized from the seeds of *Ammi majus* (*Umbelliferae* family). This compound at a single dose was tested in vitro and found to be effective and selective against hepatic CYP2A6, having a clinical utility against the metabolism of this enzyme [103,104,105]. 

### 3.7. Coumarins with Antioxidant Activity

Fraxin (Figure 20C), a coumarin derivative, demonstrated an antioxidant effect through an in vitro model in cells induced with H_2_O_2_-mediated OS showing a protective scavenging effect against free radicals at a high concentration of 0.5 mM [106]. Another coumarin that also demonstrated an antioxidant effect was esculetin (6,7-dihydroxycoumarin) through an in vitro model in Chinese Hamster lung cells (V79-4) inhibiting H_2_O_2_-induced damage of fibroblasts [107]. The pyranocoumarins grandivitin, agasilin and aegelinol benzoate, isolated from the roots of *Ferulago campestris* (Apiaceae), showed an antioxidant effect by evaluating the effect on human whole blood leukocytes and on isolated polymorphonuclear cells by chemiluminescence [108]. 

### 3.8. Coumarins as Phytoalexins

Phytoalexin is synthesized from plants in response to a fungal infection, physical damage, chemical injury, or pathogenic damage. Through various signaling mechanisms, phytoalexin inhibits foreign agents such as bacteria, insects, and even, viruses. One phytoalexin is ayapin (6,7-methylenedioxycoumarin) (Figure 20D), isolated from *Eupatorium ayapana* (Asteraceae) [109].

### 3.9. Main Biological Targets of Coumarin Derivatives According to In Vitro, In Vivo, and Structure-Activity Relationship Analyses

The therapeutic activity of coumarin compounds is largely linked to the substitution pattern in their structure. In addition, it has been reported that unsubstituted coumarins tend to be toxic when metabolized [110]. The introduction of one or several substituents has electronic effects on the structure, which is associated with the biological properties that it may possess, resulting in being very attractive in the design of new drugs, and expanding the development of bioactive molecules. In this sense, coumarins present a wide variety of biological activities as antibiotic agents, antioxidant [111], antiviral [112], antimalarial [113], antineoplastic [114], antimicrobial, anti-inflammatory, and anticoagulant.

**Figure 21:**
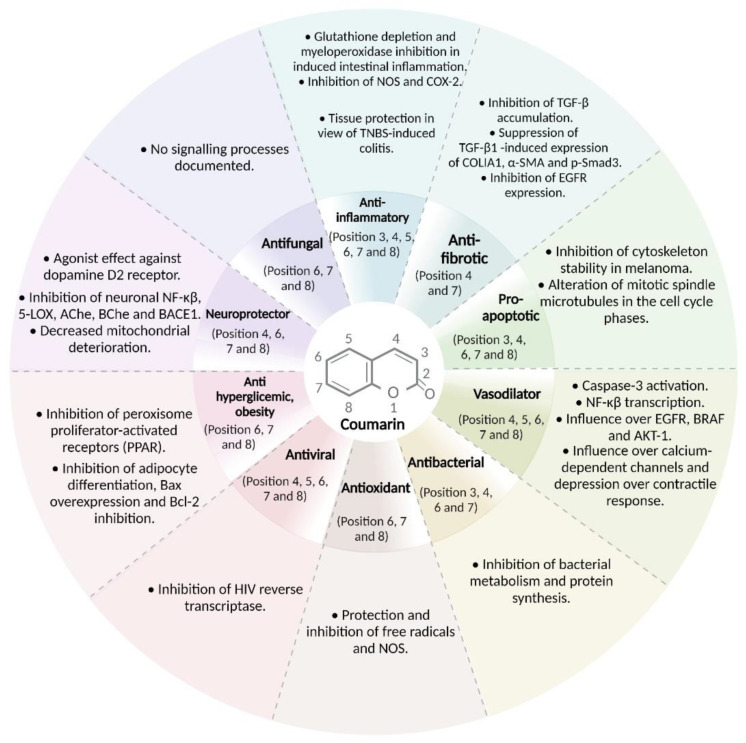
Coumarins substitution patterns and effects on the cell and molecular processes. Depending on position of the substituent (from 1 to 8) on the coumarin ring, the coumarins possess the ability to regulate biological and pathological processes, as seen pathological processes such as inflammation and/or infectious diseases. Coumarins with substituents on 3, 4, 7, and 8 positions have been the most described to have influence over these pathological processes.

The biological activity, as well as its possible therapeutic application, are related to the substitution patterns that the coumarin nucleus present. In the case of anti-inflammatory activity, in general, the derivatives substituted in positions 3 and 4 contain highly variable groups, ranging from aliphatic chains, aromatic rings to heterocycles, especially those containing nitrogen atoms (4-hydroxycoumarin, 3-(1*H*-benzo[*d*]imidazol-2-yl)-6-chloro-2*H*-chromen-2-one) [115,116]. There are few reports on substituted derivatives at position 5, when this occurs a hydroxyl or methoxy group is generally found ((*N*-(4-((2-((2-oxo-2*H*-chromen-4-yl)oxy)phenyl)-2-(piperidin-1-yl)acetamide)). On the other hand, position 6 is generally substituted with halogens such as chlorine and bromine mainly, since it is observed that they increase the activity of the molecule; positions 7 and 8 can be hydroxyl groups, aliphatic chains, aromatic systems, and heterocycles (paelanthin and aesculin) [117]. Most of the coumarins used for these type of conditions are inhibitors of the production of NO and COX-2 (Figure 21).

Regarding the coumarins used as anticoagulants (acenocoumarol, phenprocoumon, and dicoumarol) and antihypertensives (dihydrommamea), they share substituents at positions 3, 4, and 5 of the coumarin ring. Coumarins with substituents in positions 6 and 7 (scopoletin, scoparone) and substitutions 7 and 8 (visnadine) show antihypertensive activity. Dicoumarol‘s mechanism of action is that it is a selective vitamin K antagonist, while scoparone shows its effect through the activation of calcium channels.

Coumarins have an important action in infectious diseases, either as antibacterial, antifungal, or antiviral. Their function is carried out through inhibition of metabolism, reproduction, or protein synthesis. Most of the coumarins with antibacterial and antifungal activity share substituents at positions 6 and 7 of the basic coumarin nucleus (marmesin, ostruthin, phelodenol, scopoletin, among others). While ammoresinol (substituents in positions 3,4 and 7) and inophyllums (substituents in 4, 5, 6, 7, and 8) have more complex substitution patterns.

The activity of coumarins has also been tested in neurodegenerative diseases, observing that structurally coumarins have substituents in positions 6 and 7, as is the case with 6-OHDA, scopoletin and esculetin. Acting anticholineterase activity (scopoletin) or inhibiting the activation of NK-kB via 5-LOX.

The study of coumarins in the field of chronic diseases has also been developed. Esculetin, with a 6,7-substitution pattern, showed activity against obesity activity by inhibiting adipocyte differentiation. While fraxidine (6,7,8 substitution) and osthole (7,8 substitution) have hypoglycemic activity by inhibiting peroxisome proliferation-receptor activation (PPAR).

The antioxidant function of coumarins is widely reported and their substitution pattern corresponds to positions 6 and 7. Examples are fraxin, esculetin, grandivittin, agasyllin and aegelinol; which exhibit a free radical scavenging effect H_2_O_2_-mediated OS.

## 4. Cancer

Cancer is a biological and genetic alteration of the cells that make up the tissues of organs and is considered a major health problem. Cancer is a process that leads to uncontrolled cell growth and proliferation that can spread to any organ or tissue within our body. The transformed “cancerous” cells have a high multiplication rate inducing the formation of tumors in the organ involved [118,119,120,121,122,123,124,125,126].

### 4.1. Molecular Aspects of Cancer and Therapies

There are distinctive characteristic properties for each type of cancer cell; however, due to the great complexity of cancer, it is difficult to distinguish the morphological changes existing in the malignant cell, so some parameters are described: increased cell proliferation, insufficient apoptosis, altered cell differentiation, uncontrolled metabolism, genomic instability, immortalization, invasion and metastasis [127,128,129,130,131,132,133]. Each of these parameters defines the degree of severity of the type of cancer, so it is of vital importance to analyze each of them in order to obtain accurate data for cancer treatment [134,135]. An essential component to modulate is the extracellular matrix (ECM), which provides support and biochemical structure to the cell. The ECM plays important roles in cell proliferation, differentiation, and tissue homeostasis. This structure consists mainly of water, proteins, and polysaccharides. In cancer, ECM characteristics are altered because cancer cells have the ability to manipulate ECM function, leading to metastasis, which is the main cause of cancer mortality. It has been observed that, in breast cancer cell lines, there is an overexpression of ECM remodeling enzymes related to poor prognosis and a high risk of relapse [136,137]. 

In recent years, several types of molecules have been investigated that have shown demonstrable therapeutic activities; however, treatment for cancer is not yet fully defined, especially for those cancers that have been diagnosed in advanced stages and those that have spread in the form of metastasis or some other type of dissemination such as hematogenous, lymphatic system and transcellular dissemination [138,139]. Cancer therapy depends on the type of tumor, its location, its extension, and the combined administration of certain types of drugs [140]. Therapeutic modalities aim to induce apoptosis to inhibit cell proliferation [141]. However, these therapies can generate drug resistance and systemic toxicity, especially in some types of cancer such as prostate cancer [142]. In recent years, researchers have focused on the development of alternative therapies against cancer progression and invasion, targeting tumor differentiation [143], inhibition of angiogenesis [144], hormonal inhibition [145] and tyrosine kinase signaling pathways involved in cancer [146]. Despite the important discoveries made in the treatment of cancer, traditional and newer therapies such as surgery, radiotherapy, chemotherapy, targeted therapy and immunotherapy have an impact on the quality of life of patients [147].

### 4.2. Coumarins with Antineoplastic Activity

**Figure 22:**
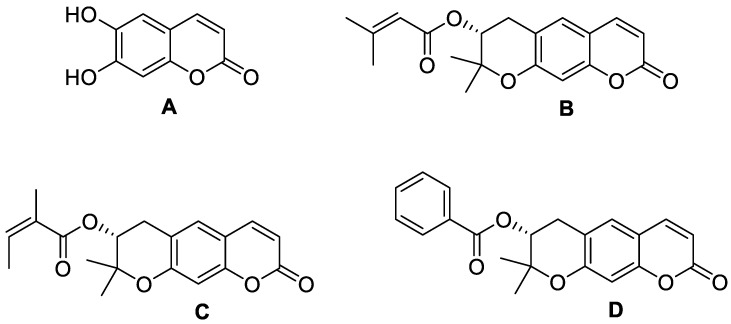
Chemical structures of esculetin (**A**), grandivittin (**B**), agasyllin (**C**), and aegelinol benzoate (**D**).

Coumarins have been tested in various cancer cell lines, demonstrating effects on various signaling pathways with an impact on the inhibition of cell proliferation or directly affecting the phases of the cell cycle and apoptotic processes [148]. Imperatorin (Figure 3B) and osthole (Figure 14B) coumarin derivatives through a series of experiments demonstrated an antineoplastic effect by inhibiting the migration and invasion of cancer cells [149]. Other derivatives such as esculetin (Figure 17) have also demonstrated an antineoplastic effect by protecting cultured primary neurons from the toxicity of *N*-methyl-d-aspartate, an anticancer compound that was injected for a prolonged period of time to determine if there were morphological changes in the neurons [150]. The coumarin derivatives grandivittin, agasyllin, aegelinol benzoate (Figure 22) and osthole (Figure 14B) obtained from the plant *Ferulago campestris* demonstrated through a series of experiments a cytotoxic effect against the A549 lung cancer cell line [151]. The anti-cancer properties demonstrated by osthole have been shown to inhibit proliferation, motility, and metastasis and to induce apoptosis and cell cycle inhibition in different cancer cell lines such as osteoblastoma in murine models [152].

#### Synthetic Derivatives of Coumarin with Antineoplastic Activity

Many coumarin derivatives, depending on the substituent, have been shown to affect signaling pathways involved in cancer development, such as the inhibition of cell cycle phases. Table 2 shows the structures and substituents and the pharmacological function they have presented as possible anticancer agents tested in various cancer cell lines.

### 4.3. Coumarin Derivatives and Their Effect on Cell Signaling Pathways in Cancer

Coumarins have cytotoxic and cytostatic effects against Hep2 cells, and they have the capacity to inhibit proliferation in lung (A549, H727), renal (ACHN), breast (MCF7) and leukemia (HL-60) cancer cell lines, showing inhibition effects on cell cycle phases, nuclear fragmentation, cell membrane loss, and even, inducing apoptosis [167,168]. 

Coumarins have impacted cancer therapy due to their affinity and specificity towards a certain therapeutic target; thus, the synthesis and development of new coumarin hybrid molecules with greater bioavailability and anticancer effects is an attractive approach. However, coumarins regulate the expression and inhibition of apoptotic proteins such as Bax and caspase-3 as well as anti-apoptotic proteins, such as Bcl-2, cell cycle regulatory proteins impacting on the inhibition of G0-G1 phases. Coumarin treatment inhibits angiogenesis and metastasis through the inhibition of vascular endothelial growth factor (VEGF) and matrix metalloproteinase (MMP) expression [169]. Coumarin also inhibits of the expression of inflammatory inhibitors, such as iNOS and COX-2, through the NF-κB, MAPK and Akt signaling pathways [170]. 

A pathway with an impact on cancer development is p53-MDM2. MDM2 (mouse double minute 2 homolog) is an important negative regulator of the tumor suppressor factor p53. Paiva et al. focused on developing a new inhibitor of p53-MDM2 interactions through nanocapsule formulations tested in MCF-7 breast cancer cell lines, analyzing the cytotoxic effect. These nanocapsule formulations were loaded with xanthone to test the anticancer effect, in addition to the incorporation of 6-coumarin, which emits a green fluorescence, showing efficacy against this signaling pathway in breast cancer adenocarcinoma [171]. 

Estrogens are relevant in the development of breast cancer, so therapies are aimed at inhibiting estrogen activity. Some coumarin derivatives, especially cinnamoyl-coumarin, were tested in estrogen-dependent breast (MCF-7) and ovarian (OVCAR) cancer cell lines [172]. These compounds are selective non-steroidal inhibitors of the 14α-hydroxysteroid dehydrogenase type 1 enzyme that has the ability to catalyze the NADPH-dependent reduction of estrogen (estradiol) [173]. Seidel et al. conducted a study in which they synthesized coumarin derivatives containing ketone groups at positions 3 and 4 of the basic coumarin nucleus. These compounds were shown to inhibit proliferation in cell lines of chronic myeloid leukemia K-562 and histiocytic lymphoma U-937 cells. In addition, there was an inhibition of histone deacetylases (HDAC), which are overexpressed in the development of cancer [174]. 

On the other hand, Yang et al. demonstrated the effect of esculetin (Figure 17) on the apoptotic signaling pathway in 3T3-L1 adipocytes, determined in adipose tissue mass involving cell death mechanisms. They tested the effect of esculetin which stimulated Bax overexpression and Bcl-2 inhibition. Inhibition of the MAPK signaling pathway, involved in the mitochondrial apoptosis-mediated pathway, was also demonstrated [175]. Ragab et al. concluded that the combination therapy of taxol, a chemotherapeutic, with esculetin has the ability to activate apoptotic signaling pathways, suggesting a new chemotherapeutic strategy. Esculetin was found to be a lipoxygenase inhibitor, showing an inhibitory effect on cell cycle progression in HL-60 cells, also tested in HepG2 hepatoma cells [176]. 

Some coumarin derivatives, such as 6-nitro-7-hydroxycoumarin, 8-nitro-7-hydroxycoumarin, and 3,6,8-trinitro-7-hydroxycoumarin, have also been tested to identify their potential effects against cancer, and have been found to have a cytotoxic effect impacting apoptosis-mediated cell death in kidney cancer cell lines [177,178]. It was demonstrated that 6-nitro-7-hydroxycoumarin has the ability to activate the MAPK signaling pathway involving p38 activation and the stress-involved SAPK protein, altering cell cycle progression [179,180]. 

Another coumarin derivative is 6-methoxy-7-hydroxycoumarin (scopoletin, Figure 8B), shown to have pro-apoptotic effects by activating caspase-3. Scopoletin was also tested in promyelocytic HL-60 cells, and was shown to induce the NF-κB transcription factor signaling pathway [181,182]. Similarly, 7,8-dihydroxy-4-methylcoumarin, another coumarin derivative, was shown to induce apoptosis in non-small cell lung cancer cell lines A-549 and leukemia (U937 and HL-60) by activating several signal transduction pathways [164]. A pro-apoptotic effect derived from activation of the JNK signaling pathways and inhibition of the ERK1-2 and PI3K/Akt pathways was demonstrated [183]. On the other hand, oxyprenylated coumarin derivatives tested in vitro on esophageal carcinoma and colorectal adenocarcinoma cell lines, showed a cytostatic and apoptotic effect [184]. 

Phytochemicals isolated from *Ferula* spp. with chemopreventive activity, among them the coumarins 7-isopentenyloxycoumarin, umbelliprenin and gercumin, were tested on ovarian (CH1), lung (A549), and melanoma (SK-MEL-28) and bladder (5637 cells) cancer cell lines demonstrating antitumor effects [163,185].

**Figure 23:**
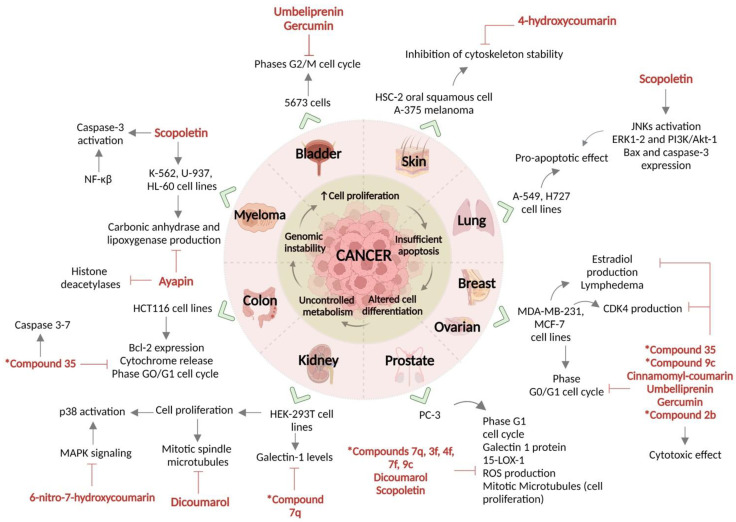
Coumarins as antineoplastic agents. Coumarins have been recently described as potent antineoplastic in different primary tumors as well as in metastatic ones. Coumarins exert a specific pro-apoptotic effect by the regulation of apoptotic pathways such as caspase, as well it has been seen as an important effect over hormonal-induced tumors such as breast and ovarium cancer, by regulating synthesis and signaling of hormones such as estradiol. *Compounds: 35 = 2-(4-(2-(tert-butoxy)-2,3-dimethyl-4-oxo-8-(quinolin-3-yl)-2,3-dihydrochromeno[3,4-*d*] imidazol-1 490 (4*H*)-yl) phenyl)-2-methylpropanenitrile; 7q = Ethyl 1-((7-((4-nitrobenzyl) oxy)-2-oxo-2*H*-chromen-4-yl) methyl) piperidine-4-carboxylate; 3f = 3-farnesyloxycoumarin; 4f = 4-farnesyloxycoumarin; 7f = 7-farnesyloxycoumarin; 9c = 7-(Diethylamino)-*N*-(4-methoxyphenyl)-2-oxo-2*H*-chromene-3-sulfonamide; 2b = Ethyl 2-((2-oxo-2*H*-chromen-6-yl)oxy)acetate.

Phenolic groups at positions 6,7 and 7,8 (7,8-dihydroxycoumarin and 6,7-dihydroxycoumarin) are essential for inducing apoptosis in cancer cell lines, tested in promonocytic leukemic cells (U-937) [186]. However, coumarin derivatives with ortho-dihydroxy substitution have shown a greater cytotoxic effect, tested in HSC-2 oral squamous cell cancer cell lines and A-375 melanoma cells [187]. A summary of the antineoplastic effects of the coumarins and the signaling pathways involved is shown in Figure 23.

### 4.4. Usefulness of Structure–Activity (SAR) Analyses of Coumarin Derivatives to Identify Molecular Targets against Cancer

The use of *in silico* experiments has revolutionized the field of research since they allow simulations or reproductions of in vivo or in vitro experiments, using mathematical models and simulation software. They make it possible to anticipate the magnitude of the responses of the system under study to variations in the intervening variables. For this purpose, new derivatives based on 6-bromo-coumarin-ethylidene-hydrazonyl-thiazolyl and 6-bromo-coumarin-thiazolyl were synthesized with the aim of creating a quantitative structure-activity relationship (QSAR) model of compounds with potential anticancer activity. The CDK4 inhibitory effect was investigated by identifying the CD4 active site and flavopiridol as a reference ligand. One of the coumarin derivatives Coumarin 2-(1-(6-bromo-2-oxo-2*H*-chromen-3-yl)ethylidene)-*N*-methyl/phenylhydrazine-1-carbothioamideshowed in molecular docking the formation of a hydrogen bond between the amino acids Asp99 and Glu144. This new coumarin-derived compound was shown to be a promising target since it showed anticancer activity against MCF-7 breast cancer cell lines by inhibiting CDK4 [160].

Other coumarin derivatives are the thiazole-hydrazone-coumarin hybrids which have shown an anticancer effect against CDK2. Cell cycle analysis showed inhibition in the G0-G1 phases. Molecular docking showed that compounds with higher cytotoxicity inhibited CDK2 involvement by generating hydrogen bonds and hydrophobic interactions with the amino acids of the CDK2 active site [188].

New coumarin derivatives (7,8-dihydroxycoumarin) have been evaluated for cytotoxicity data tested in a breast cancer cell line (MCF-7), using doxorubicin and erlotinib as positive controls. Compound *N-*(4-((2-((2-oxo-2*H*-chromen-4-yl)oxy)phenyl)-2-(piperidin-1-yl)acetamide showed the greatest effect in terms of inhibiting EGFR expression by reducing its concentration. Molecular docking analysis yielded data showing that erlotinib as a positive control interacted with EGFR tyrosine kinase, showing interaction energy with a good prognosis. ADME properties revealed high absorption in the gastrointestinal tract, making it a potential drug [189].

Phutdhawong and co-workers tested coumarin-derived compounds (coumarin-3-carboxyamides), demonstrating a cytotoxic effect on HepG2 and HeLa cancer cell lines. To demonstrate the effect, a molecular coupling was performed between one of the coumarin derivatives (2,5-difluoro benzamide derivatives) and the enzyme CK2 (protein kinase), showing a strong interaction in the active site of the enzyme, indicating that the presence of the benzamide in the basic nucleus of the coumarin provides an anticarcinogenic effect [190]. In aother study, Yuan et al. demonstrated the anticancer effect of scopoletin, a coumarin derivative, after testing it in on a lung cancer cell line (A549), using molecular docking to find new therapeutic targets and deduce the mechanism of action of this compound in a cell proliferation assay. As a result of molecular docking, it was found that the main targets of scopoletin are EGFR, BRAF and AKT-1, showing favorable interaction energies, indicating that the compound is directed towards lung cancer cells with an anticancer effect [191].

### 4.5. Adjuvant Cancer Therapy: Potential Role of Coumarins

Cancer therapies have been evolving due to the harmful effects they induce, so it is of vital importance to find adjuvant therapies that in some way help to combat the clinical manifestations caused by the development of cancer, as they can affect the post-operative recovery stage, radiotherapy, or chemotherapy, instead of only attacking the final stages of cancer [192]. Similarly, many patients are diagnosed with cancer too late for surgery. In these patients, chemotherapy and radiotherapy remain the main therapies; however, there are many side effects and complications such as myelosuppression [193], gastrointestinal tract reactions [194], cardiac [195], hepatic [196], and renal damage [197], and cancer cells have the ability to develop resistance to conventional therapies over time.

Targeted therapy is a new treatment that attacks cancer cells with greater accuracy and precision. Immunotherapy is another approach to target cancer cells, through infusions of cytokines, vaccines, and T cells, to stimulate immune cells and enhance the anticancer effect [198]. In adjuvant cancer treatments, we can mention the role played by coumarins and their synthetic derivatives [199]. Casley-Smith and coworkers synthesized coumarins from the tonka bean plant, demonstrating that they reduced lymphedema, one of the most common complications in breast cancer treatment. The study was a randomized, double-blind, placebo-controlled, crossover trial to examine the effect of coumarin (5,6-benzo-α-pyrone) in women with post-mastectomy lymphedema, demonstrating a reduction in tissue fluid with mild side effects after coumarin administration [200]. A study was conducted in which Wiadro et al. investigated the antiproliferative, chemoprotective and antiangiogenic effect of the single coumarins umbelliferone, esculin and osthole in combination with sorafenib (a specific Raf kinase inhibitor used to treat fast-accelerating fibrosarcoma) on the induction of programmed death in human glioblastoma multiforme (T98G) and anaplastic astrocytoma (MOGGCCM) cell lines. Sorafenib in adjuvant therapy with coumarins was shown to inhibit kinase expression and induce apoptosis [44].

## 5. Concluding Remarks

The coumarins and various derivatives with their substitution patterns have been very useful in the medical field and have shown important therapeutic effects in several diseases. They can attenuate the clinical manifestations of disease and have shown an impact on the life quality of patients. Their use and research have been directed against pathologies such as diabetes mellitus [98,99,100,101,102,201], obesity [202], and cardiovascular disease [63,64,65,66,67], which are considered pandemics due to the number of cases worldwide. Other diseases where coumarins and its derivatives have also had important contributions include neurodegenerative diseases such as Parkinson’s disease [203], epilepsy [204] and Alzheimer’s disease [93,94,205], infectious diseases [73,74,75,76,77,78,79,80,81,206], and liver disease [207]. Particularly, in cancer, conventional therapies have side effects in patients, impacting their life quality and therefore the use of synthetic or naturally occurring compounds have opened the way to new targeted and personalized therapies by affecting signaling pathways in which therapeutic targets are the main objective. The use of coumarins and their derivatives has shown anticancer effects in cell lines, demonstrating cytotoxicity and inhibiting signaling pathways present in cancer, showing suppression in the mechanisms of action of proteins that lead to apoptosis [208] and the inhibition of cell proliferation [209]. These studies have demonstrated that the position of the substituent in coumarin derivatives is the key point for the therapeutic effect, since it was found that the addition of substituents at carbons 3, 4, 7 of the basic nucleus generated structural changes in the molecule that confer biological effects, not only antineoplastic but also anti-inflammatory, anti-neurodegenerative, and anti-microbial, among others. Molecular docking studies have been an excellent tool to discover the interactions between derivatives and proteins present in various diseases and have functioned as molecular markers. In silico analyses have made it possible to simulate these interactions through the energies generated between the ligand and the protein, which shows the molecular coupling between the protein residues and the ligand, making it possible to demonstrate their inhibition. The most important mechanisms that influence the development of the disease have become useful tools that will serve to guide in vitro and in vivo research. Therefore, it is important to make known the role that coumarins play as potential agents in various diseases directed to specific therapeutic targets, in order for coumarins and their substituents to function as potential prodrugs and to be considered within adjuvant therapies. Since there have been no concrete investigations demonstrating that coumarins should be part of therapies, research should be directed to the search for new promising agents against a range of diseases. The prospects lead us to continue investigating the biological activities of coumarins, focusing on substituents to verify the therapeutic effect. It is of vital importance to study the mechanisms of action at the systemic level in vitro and in vivo to demonstrate that the antineoplastic effect really exists and that in the future these compounds will form a part of adjuvant therapies.

## Figures and Tables

**Table 1:** Main structures of coumarins.

Type of Coumarin	Structure	Examples of Coumarins	Reference
Single coumarin	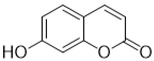 Umbelliferone (7-hydroxycoumarin)	Esculetin	[15]
		Ostrutin	[16]
		Osthole	[17]
		Novobiocin	[18]
		Coumermycin	[19]
		Umbelliferone	[20]
		Fraxidine	[21]
		Ferudenol	[22]
Furanocoumarin	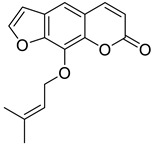 Imperatorin	Isopimpinellin	[23]
		Psoralen	[24]
		Bergaptene	[25]
		Methoxsalene	[26]
		Marmelosin	[27]
Pyranocoumarin	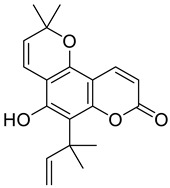 Nordentatin	1. Linear type:	[28]
		Grandivitin	[29]
		Agasyllin	[30]
		Aegelinol de Benzonatate	
		Xanthylethine	[31]
		2. Angular type:	
		Inophyllum A, B, C, E, P, G1 and G2	[32]
		Calanolide A, B and F	[33]
		(+)-Dihydrocalanolide A and B	
		Pseudocordatolide	[34]
Biscoumarin	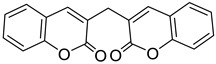 Dicoumarol	Biscoumarin	[35]
Dihydrofuranocoumarin	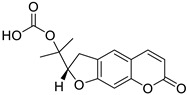 Felamidine	Anthogenol	[36]
		Marmesin	[37]
		Rutaretin	[38]
Phenycoumarins	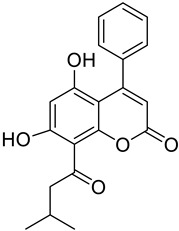 Isodispare B	Dispardiol B	[39]
		Mammea A/AB	[40]
		Disparinol D	[41]
		Disparpropylinol B	[42]

**Table 2:** Coumarin derivatives and their antineoplastic function.

Type of Cancer	Pharmacological Function	Structure	Substitute	Reference
Melanoma B16-F10	In an in vitro study, 4-hydroxycoumarin demonstrated its therapeutic effect as an antineoplastic. By inhibiting the stability of the cytoskeleton, adhesion, and motility of the melanoma cell line B16-F10.	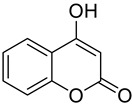 4-Hydroxycoumarin	An OH group at position 4 of the basic coumarin core	[153]
Lung	It was shown that 7-hydroxycoumarin exhibits an antiproliferative effect towards lung cancer cells (SK-LU-1, 1.3.15 and 3A5A).	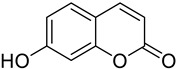 7-Hydroxycumarin	OH at position 7 of the coumarin (7-hydroxycoumarin Umbelliferone Basic nucleus)	[154]
Breast/ Ovarian/ Prostate/ Kidney	The synthesis of new 4,7-disubstituted coumarin derivatives as apoptosis-inducing agents targeting galectin-1 was performed by evaluating the cytotoxic effect on cancer cell lines: MCF7, SKOV3, PC-3, DU145, and HEK293T. Compound 7q* conjugate showed a potential antiproliferative effect against PC-3 prostate cancer cell lines by inhibiting the G1 phase of the cell cycle, reducing Gal-1 protein levels in a dose-dependent manner.	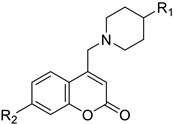 R_1_=COOC_2_H_5_ R_2_=4-NO_2_-C_6_H_4_-CH_2_ 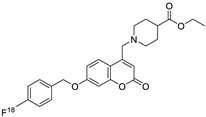	Compound: 7q* substituent R_2_ (4-NO_2_-C_6_H_4_-CH_2_).	[155]
Prostate	Overexpression of 1-lipoxygenase-1 (15-LOX-1) is considered a malignancy factor for prostate cancer. Synthetic derivatives of farnesyloxycoumarin (3f, 4f, 7f)** are potential inhibitors of 15-LOX-1, especially 7-farnesyloxycoumarin exerting cytotoxic and anticancer effects against prostate cancer PC-3 cells.	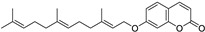 7-Farnesyloxycoumarin	Prenylated coumarins derived from farnesyloxycoumarins	[156]
Colon	Coumarin derivatives were tested in HCT116 colorectal cancer cell line, reducing its viability in a time- and concentration-dependent manner. Coumarin polysulfides accumulate in the G2-M phase of the cell cycle inducing apoptosis, decreasing balc-2, increased bax, cytochrome release, and inhibition of caspases 3-7 and cdc25C. They are considered potent antineoplastic agents.	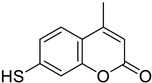 7-Mercapto-4-methyl-2H-chromen-2-one	Coumarin derivatives diallyl polysulfides (di-coumarin polysulfides) (di-coumarin polysulfides)	[157]
Lung/ Skin/ Kidney/	Several of these compounds showed antiproliferative activity. Compounds 14 and 17*** showed higher apoptotic activity. They became promising candidates for cancer treatment.	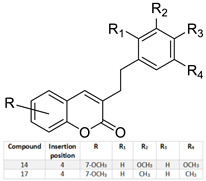	Several of these compounds showed antiproliferative activity. Compounds 14 and 17*** showed higher apoptotic activity. They became promising candidates for cancer treatment.	[158]
Breast/ Human oral epidermoid carcinoma	The cytotoxic effect of compound 9c was tested against breast cancer (MDA-MB-231) and human oral epidermoid carcinoma cell line (KB) cell lines. This compound showed antiproliferative, apoptotic activity, regulating ROS and caspase-3 levels.	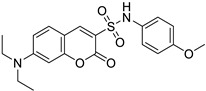 (7-(Diethylamino)-*N*-(4-methoxyphenyl)-2-oxo-2*H*-chromene-3-sulfonamide)	Coumarin derivatives with sulfonamide and amide substituents	[159]
Breast and Lung	This triad was shown to have a cytotoxic effect and a selective inhibition towards CDK4. These compounds were tested in breast cancer cell lines (MCF-7) and human lung carcinoma (A-549) and the IC_50_ was evaluated with good results.	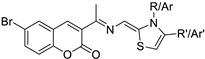 thiazole-hydrazono-coumarin	6-bromo-3-(substituted thiazolidine-2-ylidene) hydrazineylidene)-coumarin derivatives with different substituents at the 3-position of the coumarin nucleus	[160]
Bone marrow (myeloma)	This compound showed an inhibitory effect against two enzymes, carbonic anhydrase, and lipoxygenase, which are involved in the development of cancer, so this compound is considered a promising target for inhibiting these enzymes.	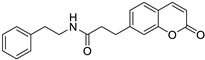 2-(2-(2-Oxo-2*H*-chromen-7-yl) oxy)-*N*-phenethylacetamide	6,7-substituted coumarins derived from 7-hydroxycoumarins	[161]
Prostate/ Melanoma/ Kidney	A coumarinic derivative 3,3’-methylenebis(4-hydroxycoumarin) “Dicoumarol” was shown to have an antineoplastic effect when tested in vitro in prostate, kidney, and melanoma cancer cell lines, as it inhibits cell proliferation by affecting mitotic spindle microtubules in the cell cycle phases.	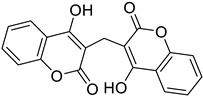 3,3’-methylenebis(4-hydroxycoumarin)	In position 3 there is a methylene bond	[162]
Bladder	7-isopentenyloxycoumarin was shown to have anticancer effects by demonstrating a cytotoxic effect against bladder cancer cell lines (5673 cells) by inducing apoptosis and inhibition of the G2/M phases of the cell cycle.	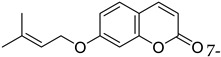 7-isopentenyloxycoumarin	Isopentenyl group	[163]
Small cell lung	7,8-Dihydroxy-4-methylcoumarin was shown to induce apoptosis tested in A549 lung cancer cell lines by activating the mitochondria-mediated caspase-dependent pathway.	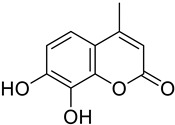 7,8-Dihydroxy-4-methylcoumarin	Methyl group at position 4 and 7,8 Hydroxy.	[164]
Breast and Colon	A new coumarinic derivative containing [3,4-*d*] imidazole-4(1*H*)-one was synthesized and its biological effect, named “compound 35”, was evaluated. It showed anticancer activity against colon cancer cell lines HCT116 and breast cancer MCF-7, affecting apoptosis and inhibiting the G0/G1 phases of the cell cycle.	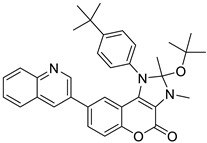 2-(4-(2-(tert-butoxy)-2,3-dimethyl-4-oxo-8-(quinolin-3-yl)-2,3-dihydrochromeno [3,4-*d*] imidazol-1 490 (4*H*)-yl) phenyl)-2-methylpropanenitrile (Compound 35)	[3,4-*d*] imidazole-4(1*H*)-one derivatives	[165]
Lung	Isofraxidin was shown to possess apoptosis-inducing activity against A549 lung cancer cell lines demonstrating its effectiveness as a potential anticancer agent.	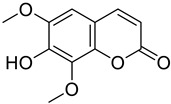 7-hydroxy-6, 8-dimethoxy coumarin (Isofraxidin)	OH group at position 7 and methoxy at positions 6 and 8	[166]

* 7q: Ethyl 1-((7-((4-nitrobenzyl) oxy)-2-oxo-2*H*-chromen-4-yl) methyl) piperidine-4-carboxylate. ** 3f: 3-farnesyloxycoumarin; 4f: 4-farnesyloxycoumarin; 7f: 7-farnesyloxycoumarin. *** Compound 14: 7-Methoxy-4-[(*E*)-2-(3,5-dimethoxyphenyl)vinyl]-2*H*-chromen-2-one; Compound 17: 4-[(*E*)-2-(3,5-Dimethylphenyl)ethyl]-7-methoxy-2*H*-chromen-2-one.
